# Religious affiliation and the division of housework over the life course

**DOI:** 10.1016/j.alcr.2025.100676

**Published:** 2025-05-17

**Authors:** Luoman Bao, Zhe (Meredith) Zhang, Roseann Giarrusso

**Affiliations:** Department of Sociology, California State University, Los Angeles, United States

**Keywords:** Housework, Division of labor, Life course, Religion, Growth curve analysis

## Abstract

A growing body of research examines changes in the division of housework over the life course, yet the influence of religion remains underexplored. Using couple dyad data from the Longitudinal Study of Generations (1988–2005) and a life course perspective, this study investigates how wives’ and husbands’ religious affiliations shape life course trajectories of the housework gender gap and individual housework time among different-sex couples in the U.S. Growth curve analyses reveal religious differences in these trajectories. Among couples where either spouse is Mormon or the wife is Catholic, the housework gender gap declined over the life course, whereas it remained stable among Protestant (mainline or evangelical), Jewish, other religious, and nonreligious couples. These declines appear to be driven by reductions in wives’ housework time over the life course among Mormon couples and increases in husbands’ housework contributions over time among couples with Catholic wives. Notably, religious disparities in the housework gender gap followed distinct patterns across life stages: the initially larger gender gap among Mormon couples and couples with Catholic wives in early life converged in midlife and became smaller than in some groups in later life. This study advances research on gender inequality in housework by highlighting the role of religious affiliation over the life course. The findings inform targeted policy interventions to support women disproportionately affected by excessive housework burdens in early life.

## 1. Introduction

Despite a narrowing gender gap in household labor in recent decades, women continue to perform significantly more housework than men (Perry-Jenkins & Gerstel, 2020). For instance, the gender gap in weekly time spent on total housework between married women and men in the U.S. remained substantial at 6.4 h in 2022–23, down from 8.4 h in 2003–05 (Milkie et al., 2025). Prolonged exposure to demanding housework or the double burden of work and domestic responsibilities can contribute to broader disadvantages for women, translating into larger cumulative economic and health inequalities in later life (Chen et al., 2019; Moen, 2016). While a growing body of research has examined changes in gender inequality in housework over the life course (Artis & Pavalko, 2003; Lam et al., 2012; Leopold et al., 2018; Nitsche & Grunow, 2016), the role of religion in shaping the stability and change in the division of housework over the life course remains largely underexplored.

As a cultural force and social institution, religion is closely intertwined with the institution of family, shaping expectations and behaviors in family life and relationships (Carroll et al., 2000; Mahoney, 2010). Religious traditions influence the division of household labor by promoting theologically grounded gender and family values through scriptural interpretations and religious teachings (Civettini & Glass, 2008; Ellison & Bartkowski, 2002; Mahoney, 2010; Voicu et al., 2009; Wilcox, 2004). Religious communities reinforce these influences through social networks that foster shared expectations and accountability for spousal roles in the household. Consequently, religion may exert a lasting influence on housework over the life course by providing normative frameworks that guide couples’ allocation of household labor across key life transitions. Additionally, religious identities are closely linked to broader family dynamics, including the timing of marriage and parenthood, family size, and household support structures. Therefore, religious affiliations may be associated with distinct trends of gender inequality in housework over the life course, with religious disparities in the gender gap evolving across life stages. However, most studies on religion and housework rely on cross-sectional data (Civettini & Glass, 2008; Ellison & Bartkowski, 2002; Gull & Geist, 2020; Voicu et al., 2009; Wilcox, 2004), which, despite their contributions, capture only a static snapshot of religious differences.

Despite offering valuable insights, prior studies leave unanswered questions about religious differences in the division of housework as life unfolds. This study draws on the life course perspective to examine the relationship between religion and the gender gap in housework among different-sex couples over the full adult life course from the perspective of both partners. Using growth curve models, we investigate how wives’ and husbands’ religious affiliations shape the trajectories of the within-couple gender gap in housework time while accounting for religious attendance and other influential factors. Additionally, we extend this analysis to individual housework time for both spouses, offering insights into the sources of changes in the gender gap shaped by religion. Drawing on data from the Longitudinal Study of Generations (LSOG) (1988–2005), with respondents born between 1902 and 1982, this study is the first to provide life course profiles of housework gender inequality from early adulthood to old age (ages 18–88) across religious groups in the U.S. This study advances life course research on housework by providing a more nuanced understanding of how gender inequality in housework changes over the life course for different religious groups. The study also contributes to research on religion and housework by identifying whether religious disparities in the division of housework persist, narrow, or widen across life stages.

## 2. Background

### 2.1. The division of household labor over the life course

The life course perspective conceptualizes experiences along the life course as “age-graded trajectories” influenced by individual life transitions and broader cultural and institutional contexts (Elder, 1994, p.5). According to its principle of *life-span development*, the division of household labor is not static but evolves as couples navigate life transitions and the ensuing shifts in bargaining powers, work demands, family roles, and social norms and expectations (Elder et al., 2003). For example, research on couples has shown that gender inequality in the division of housework slightly increases after parenthood and remains stable in the following years (Nitsche & Grunow, 2016). As couples move into later stages of family life when children are older, gender inequality in housework declines over time (Lam et al., 2012). Similarly, research tracking long-term trends in housework time for individuals reveals that women’s housework time culminates in young adulthood and gradually declines, while men’s housework time remains consistently lower until midlife, followed by a slight increase in older age, leading to a convergence in the gender gap over the life course (Leopold et al., 2018).

Three established perspectives – time availability, relative resources, and gender ideology – explain changes in the division of housework over the life course (Bianchi et al., 2000; Coltrane, 2000; Geist & Ruppanner, 2018; Lachance-Grzela & Bouchard, 2010). According to the time availability perspective, as women’s paid work hours increase, they reduce housework (Artis & Pavalko, 2003), while their husbands increase their share of stereotypically female housework, such as grocery shopping, washing dishes, and cleaning, that are often more time-consuming and less desirable (Cunningham, 2007). In later life, husbands’ housework contributions often rise after retirement (Leopold & Skopek, 2015). Children in the household also indicate time-related demands and availability. The gender gap in housework widens with the addition of young children (Artis & Pavalko, 2003; Craig & Mullan, 2010) but narrows as children grow up and leave home (Craig & Sawrikar, 2009; Schulz & Raab, 2023).

The relative resources perspective suggests that economic resources translate into bargaining power in housework allocation, allowing the higher-earning spouse to negotiate out of undesirable chores. Husbands’ higher earning power is linked to wives’ greater housework contributions (Lam et al., 2012), and wives’ relative earnings increase husbands’ participation in stereotypically female housework (Cunningham, 2007). Moreover, increases in wives’ relative, or more importantly, absolute economic resources reduce their housework over time (Artis & Pavalko, 2003; Evertsson & Nermo, 2007; Killewald & Gough, 2010; Sullivan & Gershuny, 2016) by enabling them to forego or outsource some tasks (Killewald & Gough, 2010). Combining time availability and relative resources perspectives, women who reduce labor force participation for caregiving often experience income loss and career disruptions, which may limit their bargaining power in housework allocation both short-term and over the life course.

The gender perspective underscores the gendered meanings and norms tied to housework, emphasizing its allocation as a process of “doing gender” (West & Zimmerman, 1987, p.126) and an enactment of gender roles and relations formulated by spouses’ gender ideologies (Bianchi et al., 2000; Geist & Ruppanner, 2018). Both partners’ egalitarian gender ideologies are associated with a more egalitarian division of housework over time (Cunningham, 2005; Nitsche & Grunow, 2016). Gender ideologies can also change across the life course (Cunningham, 2008), with wives’ increasing egalitarianism linked to reduced gender gaps in housework (Artis & Pavalko, 2003) and increased husbands’ participation in stereotypically female housework (Cunningham, 2007).

### 2.2. Religion and housework over the life course

While established perspectives on time availability, relative resources, and gender ideology offer valuable insights into changes in the division of household labor over the life course, the role of religion as a cultural and institutional force remains understudied. Religion is closely linked with these perspectives and may jointly shape housework patterns. Specifically, religion influences individuals’ gender roles and attitudes, which in turn affects couples’ time allocation and resource accumulation across the life course. Existing work on religion and housework is limited and primarily based on cross-sectional data. Little research has explored how religious affiliation shapes the division of household labor over the life course. To address this gap, we first discuss how religion is associated with the gender gap in housework and then turn to religion’s role in shaping the trajectory of this gap over the life course.

#### 2.2.1. Religious affiliation and gender inequality in housework

Historically rooted in religious scripture, gender and family values have been developed, disseminated, and, in some cases, either perpetuated or adapted through the interpretations of religious leaders, religious teachings and organizational activities, and popular religious literature (Civettini & Glass, 2008; Ellison & Bartkowski, 2002). These processes guide housework behaviors by establishing diverse or similar norms and expectations around gender and family roles for religious communities (Voicu et al., 2009; Wilcox, 2004). Believers often seek divine guidance and draw on religious wisdom to navigate their roles in family life and domestic labor (Mahoney, 2010) and perceive the fulfillment of those theologically grounded expectations in their gender and family roles as a validation of the sacred nature of their family life and relations (Mahoney et al., 2003). Therefore, patterns of household labor division may be similar among some religious affiliations and differ among others.

Conservative (i.e., fundamentalist and evangelical) Protestantism emphasizes biblical inerrancy but becomes segmented in its contemporary discourses on gender roles. Traditionalist evangelicals endorse distinct gender roles, viewing men as providers and household leaders responsible for masculine chores, while women are primarily responsible for housework and caregiving (Bartkowski, 1999; Ellison & Bartkowski, 2002). These views draw on biblical interpretations, including Genesis, which positions Adam as the provider and Eve as his helper, as well as Pauline references and Proverbs 31, which extol the ideal wife as selflessly dedicated to domestic obligations (Ellison & Bartkowski, 2002). In contrast, egalitarian evangelicals support more flexible gender roles and an equitable sharing of housework, interpreting biblical guidance on wives’ domesticity as context-specific to the early church rather than a timeless prescription and framing biblical teachings on husbands’ Christ-like sacrificial love for their wives as a foundation for gender equality in the home (Ellison & Bartkowski, 2002). Conservative Protestantism also espouses traditional familism, positioning the family as sacred and central to individuals’ lives and housework as a meaningful enactment of faith and family values (Civettini & Glass, 2008; Wilcox, 2004). Since the 1970s, conservative Protestant discourse has increasingly encouraged men to take on greater familial obligations, both as engaged fathers and as husbands who alleviate their wives’ daily burdens through routine housework participation (Bartkowski, 1999; Civettini & Glass, 2008). This renewed admonition on men’s dual responsibilities in breadwinning and familial involvement peaked in the mid-1990s with the rise of organizations like the Promise Keepers (Civettini & Glass, 2008).

Mainline Protestantism, while sharing theological roots with conservative Protestantism, differs in its progressive biblical interpretations and modernizing agenda to adjust church teachings to broader societal shifts (Wilcox et al., 2004). Mainline Protestant churches generally embrace more egalitarian gender roles and promote a form of familism that encourages men’s involvement in domestic labor (Wilcox, 2004). Empirical findings on housework differences between conservative (i.e., evangelical) and mainline Protestants are mixed. Some studies suggest that mainline Protestant men with children perform more housework than their conservative counterparts (Wilcox, 2004). However, other research finds only modestly greater inequality in wives’ housework time and gender segregation of household labor among conservative Protestant couples compared to nonevangelical counterparts (Ellison & Bartkowski, 2002) and no significant difference in men’s housework contributions between the two denominations (Civettini & Glass, 2008).

The Catholic Church upholds a traditional family discourse situated between the traditionalism of conservative Protestantism and the liberalism of mainline Protestant family discourse (Wilcox et al., 2004). Within the Catholic Church in the U.S., divisions between conservative and liberal Catholics have grown regarding gender roles, with conservative Catholic women often espouse motherhood and child-rearing as central to women’s roles (Bulanda, 2011), while liberal Catholics advocate for more equitable gender roles in both public and private spheres (Manning, 1997). In Europe, the Catholic Church promotes more conservative gender roles than Protestant churches, contributing to greater gender inequality in housework in Catholic-majority countries than Protestant ones (Voicu et al., 2009). A global study found that Catholic men exhibit a more traditional division of labor in stereotypically female housework tasks, though their total time spent on housework does not differ significantly from nonreligious men (Gull & Geist, 2020).

Similar to conservative Protestantism, Mormon doctrine places a strong emphasis on the sacred nature of family. Over the last century, Mormon church leaders have increasingly stressed the paramount importance of family in Mormons’ lives and advocated for a dichotomized division of labor, with men as breadwinners and women as homemakers (Heaton et al., 1994). Consequently, Mormons tend to endorse more traditional gender ideologies and family values, adhering to a more gendered division of labor – husbands as financial providers and wives as homemakers and caretakers – compared to conservative Protestants, Catholics, and nonreligious individuals (Carroll et al., 2000; Heaton et al., 1994). Research on housework among Mormons remains sparse, but one study reveals greater gender inequality in housework time among Mormons compared to non-Mormons in the U.S. This disparity is largely driven by Mormon women’s significantly higher time investment in housework than other women, despite Mormon men contributing slightly more than men from other groups (Heaton et al., 1994).

Jewish individuals generally embrace more egalitarian gender roles than other religious groups (Bulanda, 2011; Fishman, 2014). One of the foundations of gender egalitarianism in Jewish communities arises from their gender role expectations that allow men to be nurturing and emotionally expressive and women to be resilient and competent (Hartman, 2017). Additionally, familial values in Judaism, besides its emphasis on the centrality of the family, also stress family contributions from both women and men. It has been a tradition that Jewish women’s family roles extend beyond domestic tasks by including providing for the family economically, while Jewish men are expected to actively engage in parenting and meet their wives’ needs (Hartman, 2017). Furthermore, contemporary Jewish families have narrower gender gaps in socioeconomic status than the general U.S. population, leading to the endorsement of greater flexibility in gender roles to strengthen family resilience (Fishman, 2014). These factors have fostered the rise of “spousal partnership” in American Jewish families, characterized by dual-earner arrangements, shared household responsibility and authority, and collaborative negotiation of housework and caregiving (Fishman, 2014, p.103). Although Jewish women still perform more housework than men, empirical research indicates that Jewish men engage in a more egalitarian division of household labor than nonreligious men (Gull & Geist, 2020).

Taken together, examining the relationship between religious affiliation and the division of housework is important as religious traditions construct and legitimize gender roles and family values, influencing expectations and behaviors around housework. However, most research on religion and housework in the U.S. has focused on comparisons between evangelical and mainline Protestants, with relatively few studies exploring housework patterns across a broader range of religious groups and how religious disparities evolve across life stages.

#### 2.2.2. Religious affiliation and changes in gender inequality in housework over the life course

Theoretical perspectives on cultural influences in durable inequality suggest that socioculturally constructed identities and narratives guide labor divisions and legitimize inequalities between social groups (Valentino & Vaisey, 2022). Accordingly, early socialization into religious identities and values shapes how couples establish housework arrangements, anchoring their practices in religiously prescribed gender norms and family expectations. As couples navigate key family life transitions that alter housework demands and sources of support, religious discourses and teachings continue to provide normative frameworks that guide and justify continuity or adjustment in housework allocation. Thus, religion exerts a lasting influence on the division of housework over the life course.

As religious couples tend to adhere to the gender roles and family values prescribed by their faith when adjusting housework arrangements in response to life transitions, the resulting division of housework is likely to remain aligned with their religious tradition’s expectations. Therefore, different religious affiliations may shape the trajectory of gender inequality in housework over the life course in distinct ways.

After the transition to parenthood, couples affiliated with liberal religious denominations that support egalitarian gender roles are likely to continually uphold these values rather than reverting to traditional gender roles. They may redistribute additional housework in ways that sustain equitable practices and reflect shared family responsibilities. Similarly, men in conservative denominations that increasingly espouse flexible gender roles and greater male involvement in family life may respond to religious expectations by increasing their housework participation after parenthood in order to ease their wives’ domestic workload. Once these couples reach a new equilibrium in housework allocation, the gender gap in housework may remain largely unchanged. As couples transition into later life stages, such as children growing older or retirement, religiously grounded norms of gender equality or male familial obligations may continue to reinforce an equitable division of housework, keeping the gender gap relatively stable at a lower level.

In contrast, among couples affiliated with conservative religious denominations that emphasize distinct gender roles – women as primary caregivers and homemakers and men as financial providers – religious discourses often amplify these traditional divisions during early life stages of parenthood and family expansion. Increased housework demands from childrearing, especially in time-intensive traditionally feminine tasks like cooking, cleaning, and laundry, tend to fall disproportionately on women, legitimized by religious discourses that uphold dichotomized gender roles. Demanding housework and childcare responsibilities may lead women to reduce their labor force participation, resulting in lower earnings (Perry-Jenkins & Gerstel, 2020), while husbands increase their paid work to fulfill financial responsibilities. These time and economic dynamics can further reinforce traditional gender roles within the household. Additionally, conservative religious identities are closely tied to other family behaviors, including early parenthood and higher fertility. For instance, Mormons tend to marry and have children earlier and have higher fertility rates than both the national average and most other religious groups (Heaton et al., 1994; Pew Research Center, 2015; Uecker & Stokes, 2008). These early life transitions can disrupt women’s education and career trajectories, limiting their opportunities for economic independence and diverse role identities, thus entrenching greater gender inequality in household labor (Coltrane, 2000). All these mechanisms may collectively exacerbate gender inequality in housework for these couples during early life stages.

However, the gender gap in housework for these couples may narrow in midlife as children grow older or leave home, reducing women’s housework responsibility. Later-life family transitions may further reshape housework arrangements. Religious parents often socialize their children into gender roles and family values aligned with the discourses and teachings of their denominations. In families with conservative religious affiliations, adult daughters are likely to be socialized into traditional gender and family roles, and under the familistic orientations of these families, they may provide additional housework support to alleviate their mothers’ burden. Research has suggested that gendered patterns of housework division are likely transmitted intergenerationally, and daughters often contribute more to household tasks (Cordero-Coma & Esping-Andersen, 2018). Meanwhile, as financial pressures ease with children’s independence or as retirement approaches, men in conservative religious families may scale back work commitments and increase their housework participation. Together, these family life transitions may contribute to a continued decline over time in the gender gap in housework.

### 2.3. The present study

In this study, we draw on the life course perspective to examine how religious affiliations shape the trajectory of the within-couple housework gender gap over the life course. We hypothesize that different denominational affiliations are associated with distinct trajectories (H1). Specifically, couples affiliated with conservative religious traditions that uphold distinct gender roles are expected to experience a declining trajectory, with substantial gender gaps in early adult life that gradually diminish over time. In contrast, couples affiliated with religious traditions that emphasize flexible or egalitarian gender roles and men’s greater familial involvement are expected to maintain relatively stable trajectories with smaller gender gaps. Consequently, when comparing these trajectories, we expect religious disparities in the housework gender gap to evolve at different life stages (H2). Religious disparities in housework gender gap may be most pronounced in early life, with couples in traditions emphasizing distinct gender roles experiencing significantly higher levels of gender inequality. These disparities may narrow and converge in midlife and later life as the gender gap among these couples declines to levels comparable to others.

In addition, we hypothesize that the declining trajectory of the housework gender gap may result from either decreases in wives’ housework time or increases in husbands’ housework time over the life course (H3). Changes in the gender gap can stem from shifts in either spouse’s housework contributions (Bianchi et al., 2000; Coltrane, 2000). To investigate the sources of change, we examine how religious affiliations are associated with the trajectories of wives’ and husbands’ absolute housework time. This approach provides deeper insights into the mechanisms driving changes in the gender gap trajectory.

## 3. Method

### 3.1. Data

We used five waves of data from the Longitudinal Study of Generations (LSOG) over 17 years, from 1988 to 2005. Since 1971, the LSOG has traced respondents of different generations in about 350 multigenerational families randomly drawn from a list of 840,000 members of a California Health Maintenance Organization (for more details, see Bengtson et al., 2009). The survey offers an unprecedented scope of couple data on household labor and religious characteristics over the life course, with information reported by both spouses. We set the 1988 wave as wave 1 for the current analysis because some covariates used in our study were not asked in earlier waves. Waves 2–5 were based on subsequent data collected in 1994, 1997, 2000, and 2005 (waves 1–5 hereafter). We excluded the 1991 wave because it did not ask about household labor. After removing 7 respondents who did not report their birth year, the original data contains 1482, 1688, 1720, 1954, and 1796 respondents in waves 1–5. In each wave, we restricted the sample to respondents currently married or cohabiting and living with their spouse or partner (345, 574, 609, 698, and 524 respondents were deleted, respectively, in each wave). We then matched respondents who were different-sex couples with both spouses participating in the survey wave, forming 373, 356, 353, 436, and 386 couple dyads, respectively, in waves 1–5, yielding 1904 dyad-year observations. This couple dyad data structure allowed us to analyze the gendered division of household labor within couples and investigate how it is influenced by the characteristics of either member of the couple.

Wife’s and husband’s weekly housework hours, income and work hours have about 10–15 % missing data, while other variables have 5 % or less. The higher rate of missingness in these variables may stem from difficulties estimating weekly hours for housework or paid work, or privacy concerns about reporting income or time use. Participants might also fear judgment if they perceive their responses deviate from social norms or expectations. For instance, couples where husbands contribute minimally to housework may be more likely to leave housework time questions unanswered (Coltrane, 2000).

We addressed missing data using multiple imputations via chained equations (MICE) in Stata. The imputation model included all variables in the analysis, including the dependent variables, and 20 imputations were performed to ensure stable parameter estimates. Following the multiple imputation then deletion (MID) approach, which has been shown to yield unbiased estimates (Von Hippel, 2007), we excluded observations with imputed values for the dependent variable (housework gender gap) in subsequent growth curve analyses. This resulted in an analytical sample of 1487 dyad-year observations from 661 unique couple dyads. We conducted a robustness analysis by excluding observations with missing values in any variable. Results from the complete-case data were consistent with those from the imputed data, which supports the validity of the imputation process and the reliability of our findings.

### 3.2. Measures

#### Dependent variables.

In each wave, respondents reported the total hours per week they spent on housework as a single estimate. To guide responses, the survey provided examples such as cleaning, shopping, keeping track of money and bills, doing repairs or yard work, and cooking to help respondents consider the full scope of household chores. We analyzed three dependent variables. The *housework gender gap* measures the within-couple difference in housework time, calculated as the wife’s weekly housework hours minus the husband’s. *Wife’s housework hours* and *husband’s housework hours* capture the amount of time each spouse spends on housework per week. Table 1 presents descriptive statistics for all variables in the study. On average, wives spent 23.22 h per week on housework, twice as much as husbands, resulting in a housework gender gap of 11.31 h (Table 1).

There has been extensive discussion on measuring the division of household labor in housework research. Both a difference measure, as used in our study, and a proportional measure – the percentage of the couple’s total housework hours contributed by the wife or the husband – are commonly used and have been demonstrated as valid approaches to capture within-couple gender inequality in housework (Bianchi et al., 2000; Coltrane, 2000; Voicu et al., 2009). The difference measure has an advantage over the proportional measure because separate analyses of wives’ and husbands’ housework hours as dependent variables could clarify how independent variables influence the two exact components of the difference measure, enabling a more precise understanding of the sources of change in housework gender gap (Bianchi et al., 2000). Another strength of our housework measures is that they are obtained from self-reports by both spouses, which may yield more accurate estimates than relying on one spouse to report for both.

#### Independent variables.

Key variables of interest included wives’ age and both spouses’ religious affiliation and attendance. We measured *wives’* and *husbands’ religious affiliation* by collapsing 79 religious denominations in the responses into seven categories based on established classification frameworks in the literature (Hwang et al., 2018; Steensland et al., 2000): Mainline Protestant, Evangelical Protestant, Catholic, Jewish, Mormon or Church of Latter Day Saints (Mormon), other, and none.

Religious service attendance, an indicator of religiosity, may also shape housework behaviors and the associated gender gap. Frequent attendance may be part of a conventional lifestyle that includes traditional gender roles and housework practices (Wilcox, 2002). Regular church attendance also increases exposure to religious discourse and teachings on gender and family (Voicu et al., 2009), which can reinforce adherence to religious tenets and enhance their influence on family behaviors. Moreover, frequent attendance may heighten the social control individuals experience within their religious community over family behaviors (Voicu et al., 2009). While research offers mixed findings on the effects of religious attendance – ranging from no association with housework division (Voicu et al., 2009; Wilcox, 2004) to a positive impact of husbands’ attendance on their housework time (Gull & Geist, 2020) – we control for it to better isolate the distinct effects of religious affiliation. *Wives’* and *husbands’ religious attendance* were derived from the survey question “How often do you attend religious services these days?” Responses of “never” and “once a year or so” were grouped into 1 = infrequent attendance; “several times a year” and “about once a month” were recoded into 2 = moderate attendance; and “once a week or so” and “more than once a week” were combined into 3 = frequent attendance. The grouping of adjacent categories was informed by their similar coefficients in the analytical model, enhancing interpretability while preserving the key patterns in the data.

Wives’ and husbands’ religious affiliation and attendance were treated as time-varying variables in the models due to considerable changes observed over the life course in the data, consistent with literature suggesting that individuals’ religious characteristics can shift over time (Hwang et al., 2022; Silverstein & Bengtson, 2018). Fig. 1 in the Appendix presents within-couple stability and change in wives’ and husbands’ religious characteristics across waves among couples interviewed in at least two waves. About a quarter of wives and husbands experienced changes in religious affiliations, and about 30 percent of them reported changes in religious attendance over time.

We analyzed the influences of wives’ and husbands’ religious characteristics in separate models to examine the effects of each spouse’s religious attributes while mitigating multicollinearity concerns due to their strong correlations. Specifically, we found high associations between wives’ and husbands’ religious affiliation (Cramér’s V = 0.72) and religious attendance (Kendall’s tau-b = 0.77).

#### Control variables.

We controlled for wives’ and husbands’ race/ethnicity, health status, and indicators of their resources, time availability, and gender ideology, as these factors may influence the housework gender gap as well as wives’ and husbands’ individual housework time (Artis & Pavalko, 2003; Bianchi et al., 2000; Geist & Tabler, 2018; Lam et al., 2012; Nitsche & Grunow, 2016; Perry-Jenkins & Gerstel, 2020). *Race/ethnicity* was coded as 1 = White and 0 = Other, as only 6.9 % of wives and 7.5 % of husbands identified as non-White. *Self--reported health* was categorized as 1 = poor/fair, 2 = good, and 3 = excellent, with poor and fair combined due to small sample sizes. *Education* was measured on an 8-point scale in the survey (1 = 8th grade or less to 8 = post-graduate degree) and recoded into four categories in the study based on the data distribution and similar coefficients among adjacent categories. *Income last year* was measured on the survey’s original scale from 0 ($0) to 12 ($110,000 or more). Time availability was assessed through the *number of children in the household* and both spouses’ *work hours per week*. *Gender ideology* was calculated as the mean score of six items (with item five reverse-coded), where higher scores indicated more egalitarian views. The items, adapted from Levinson and Huffman’s (1955) scale, were measured on a 4-point scale (1 = strongly agree to 4 = strongly disagree). They included: (1) Some equality in marriage is a good thing, but by and large, the husband ought to have the main say in family matters; (2) It goes against nature to put women in positions of authority over men; (3) The increase in the number of women who work has led to a decline in the quality of family life; (4) Women who want to remove the word ‘obey’ from the marriage service do not understand what it means to be a good wife; (5) The women’s liberation ideas make a lot of sense to me; and (6) A woman who places more importance on her career than on being a mother is denying her true nature. The scale demonstrated strong reliability, with an average Cronbach’s alpha of 0.82 for both wives and husbands across waves. To address attrition-related bias due to nonresponse and marital changes, we also controlled for dummy variables indicating whether couples were *lost to follow-up*, *divorced/separated*, or *widowed* across the five waves.

### 3.3. Analytic strategy

The study used growth curve models to predict the division of household labor over the life course as a function of wives’ and husbands’ religious characteristics. Growth curve models have several advantages (Rabe-Hesketh & Skrondal, 2012). By allowing a random intercept and a random coefficient of age, we estimate trajectories of the housework gender gap (i.e., patterns of change in gender inequality in household labor over the life course) and examine how these trajectories vary systematically due to inter-couple differences and intra-couple changes, as well as randomly. By including time-varying covariates at level 1, the model estimates represent either comparisons among different couples or among repeated observations of the same couple over time. Allowing a couple-specific random intercept at level 2 considers the effects of omitted couple characteristics or unknown heterogeneity at the couple level (e.g., underlying gender relations in the household). Growth curve models also permit data to be unbalanced across time because the estimation of trajectories is irrespective of the number of waves. The two-level linear growth curve models are specified as follows:

Level 1 model:

(1) $$Y_{tj}=\beta_{0j}+\beta_{1j}AgeC_{tj}+\beta_{2j}X_{tj}+\beta_{3j}AgeC_{tj}\times X_{tj}+\beta_{4j}Z_{4tj}+\ldots +\beta_{kj}Z_{ktj}+r_{tj}$$

Level 2 models:

(2) $$\beta_{0j}=\gamma_{00}+\gamma_{01}W_{1j}+\ldots +\gamma_{0k}W_{kj}+u_{0j}$$

(3) $$\beta_{1j}=\gamma_{10}+u_{1j}$$

At level 1, the analytical time metric was the wife’s age, centered on the grand sample mean ($AgeC_{tj}$). Wives’ age had no missing values in the sample and was grand-mean centered before MID. In the sample, wives’ ages ranged from 18 to 88, with a mean of 51.92 years. Therefore, the intercept represented the within-couple housework gender gap at the mean wife’s age of 52 years. The housework gender gap ($Y_{tj}$) for couple j at time t was first modeled as a linear function of the wife’s age ($AgeC_{tj}$). Preliminary analyses indicated that the quadratic term of age was non-significant, suggesting that linear trajectories more accurately captured changes in the housework gender gap with age. Thus, the quadratic term was excluded from the final models. Next, we added the time-varying independent variable ($X_{tj}$) of religious affiliation and all time-varying controls ($Z_{4tj}\ldots Z_{ktj}$). To examine whether trajectories of the housework gender gap varied by religious affiliation, we included an interaction term between affiliation and age.

The intercept ($\beta_{0j}$) and age slope ($\beta_{1j}$) are couple-specific, which are responses of the level-2 models. Equations at level 2 introduce randomness, estimating heterogeneity across couples in the intercept and slope of housework gender gap trajectories. Time-invariant characteristics of the couple dyad ($W_{1j}\ldots W_{kj}$), such as race/ethnicity and attrition status, were included only in the equation for the intercept because this study does not focus on cross-level interactions between these variables and age.

The analyses proceeded in several steps. First, we began with an unconditional growth curve model and sequentially added control variables and religious characteristics (affiliation and attendance) to assess the average trajectory of the housework gender gap. Next, we tested interactions between age and religious affiliation to determine whether the housework gender gap trajectory differed significantly among religious affiliations. The main effect of religious affiliation reflects its effect on the intercept of the trajectory (i.e., the average housework gender gap at the mean age of 52), while the interaction effect captures its effect on the slope of the trajectory (i.e., the rate of change in the housework gender gap with age). Preliminary analyses indicated that the effects of religious affiliation remained robust after including religious attendance. Therefore, we present the full models that control for attendance. Finally, to interpret the effects of religious affiliation on the gender gap trajectory, we examined the same interaction effects in models where wives’ and husbands’ housework hours were the dependent variables. Wives’ age (centered at 52 years) served as the time metric across all growth curve models. This approach ensures that results across models with different sets of predictors are directly comparable when interpreting at the same reference age of wives (52 years). It also aligns with the study’s focus on understanding how the housework gender gap evolves over the wives’ life course.

## 4. Results

Results from the growth curve analyses are presented in Tables 2 and 3. Table 2 shows the unconditional model (Model 1a) and the model with control variables (Model 1b) predicting the housework gender gap. Models 1c and 1e in Table 3 build on Model 1b by adding wives’ and husbands’ religious characteristics, respectively. For brevity, we refer to couples based on one spouse’s religious affiliation, using terms like “Mormon wives” for couples where the wife is Mormon and “Mormon husbands” for those where the husband is Mormon. “Mormon couples” refers to couples where either spouse is Mormon. This naming convention applies to all religious and nonreligious groups discussed in this study.

Model 1a shows that, on average, the housework gender gap slightly decreased over the life course by 0.13 h per year of age when no covariates were included. However, this age effect became non-significant after accounting for control variables and wives’ or husbands’ religious characteristics (Models 1b, 1c, and 1e), indicating that the average trajectory of the housework gender gap remained stable with age and that any observed changes in the gender gap with age may be explained by other significant covariates in the models. Specifically, wives’ higher incomes and longer work hours were associated with a smaller gender gap at the mean age of 52, whereas husbands’ higher incomes, longer work hours, and having more children in the household were linked to a larger gap (Model 1b).

Wives’ religious affiliation and attendance were not significantly associated with the housework gender gap at age 52 (Model 1c). However, husbands’ religious affiliation and attendance showed significant associations (Model 1e). Compared to Mormon husbands, Catholic and Jewish husbands had smaller housework gender gaps by 6.11 and 6.81 h, respectively, at age 52, indicating less gender inequality in household labor. These significant associations, however, disappeared after adding the interaction between religious affiliation and age (Model 1f, Table 3), suggesting that the association between husbands’ religious affiliation and housework gender gap may vary with age and cannot be fully captured by the main effects alone, which assume constant religious disparities over the life course.

Husbands with moderate religious attendance exhibited a smaller housework gender gap by 3.36 h, on average, at age 52 compared to infrequent attendees, while frequent attendance showed no significant effect (Model 1e). The findings are partially consistent with cross-sectional research showing a positive association between men’s religious attendance and housework time (Gull & Geist, 2020). However, since the effect of moderate attendance existed while accounting for religious affiliation, this suggests that its role in reducing the gender gap may not be strongly tied to affiliation-specific religious teachings. If the housework gender gap were influenced by religiosity or reinforcement of gender roles in religious discourse through service attendance, we would expect frequent attendance to have an effect, which was not observed. Unlike frequent attendance, moderate attendance (several times a year to about once a month) may primarily reflect family-oriented motivations. Husbands in this group might attend services to accompany their wives or fulfill family obligations. Religious attendance may be perceived as a structured opportunity for them to invest more time in family activities (Mahoney, 2010), signaling their greater emphasis on family life and responsiveness to family needs. Consequently, they may be more willing to share housework responsibilities. Additionally, moderate attendees may be more active in their daily lives overall than infrequent attendees, which could further contribute to their greater involvement in housework.

To examine whether housework gender gap trajectories vary by wives’ religious affiliation, Model 1d in Table 3 extends Model 1c by including the interaction between wives’ religious affiliation and age. While wives’ religious affiliation was not significantly associated with the gender gap in housework at age 52, significant interactions indicate that trajectories of the housework gender gap differed by wives’ religious affiliation.

Fig. 1 visualizes the interaction effects, illustrating the predicted trajectories of the housework gender gap for wives of different religious affiliations. These predictions are based on all coefficients in Model 1d, with other covariates held at their means for continuous variables and modes for categorical variables. For Mormon wives, the housework gender gap decreased significantly by 0.49 h for each additional year of age (Model 1d). Catholic wives also exhibited a significant (p < 0.05) but less steep decline in the gender gap trajectory, at 0.21 h per year (supplementary analyses with each affiliation as the reference category not shown). The trajectories for wives with mainline Protestant, evangelical Protestant, Jewish, other, or no religious affiliation significantly differed from that of Mormon wives, as their housework gender gaps did not significantly change with age.

Additional tests at each age point in Fig. 1 revealed that disparities in the housework gender gap by wives’ religious affiliation follow different patterns across life stages (results available upon request). In early life (ages 18–38), Mormon wives had significantly larger gender gaps than all other wives, while Catholic wives had larger gender gaps than mainline Protestant wives between ages 18 and 28. In midlife, no significant differences in the gender gap were observed across religious groups. In later life, the gender gaps for Mormon and Catholic wives became significantly smaller than those of mainline Protestant, evangelical Protestant, and nonreligious wives. This pattern emerged around age 68 for Mormon wives and age 58 for Catholic wives, persisting through age 88. Jewish wives also had smaller housework gender gaps than nonreligious wives between ages 48 and 78.

**Fig. 2.**
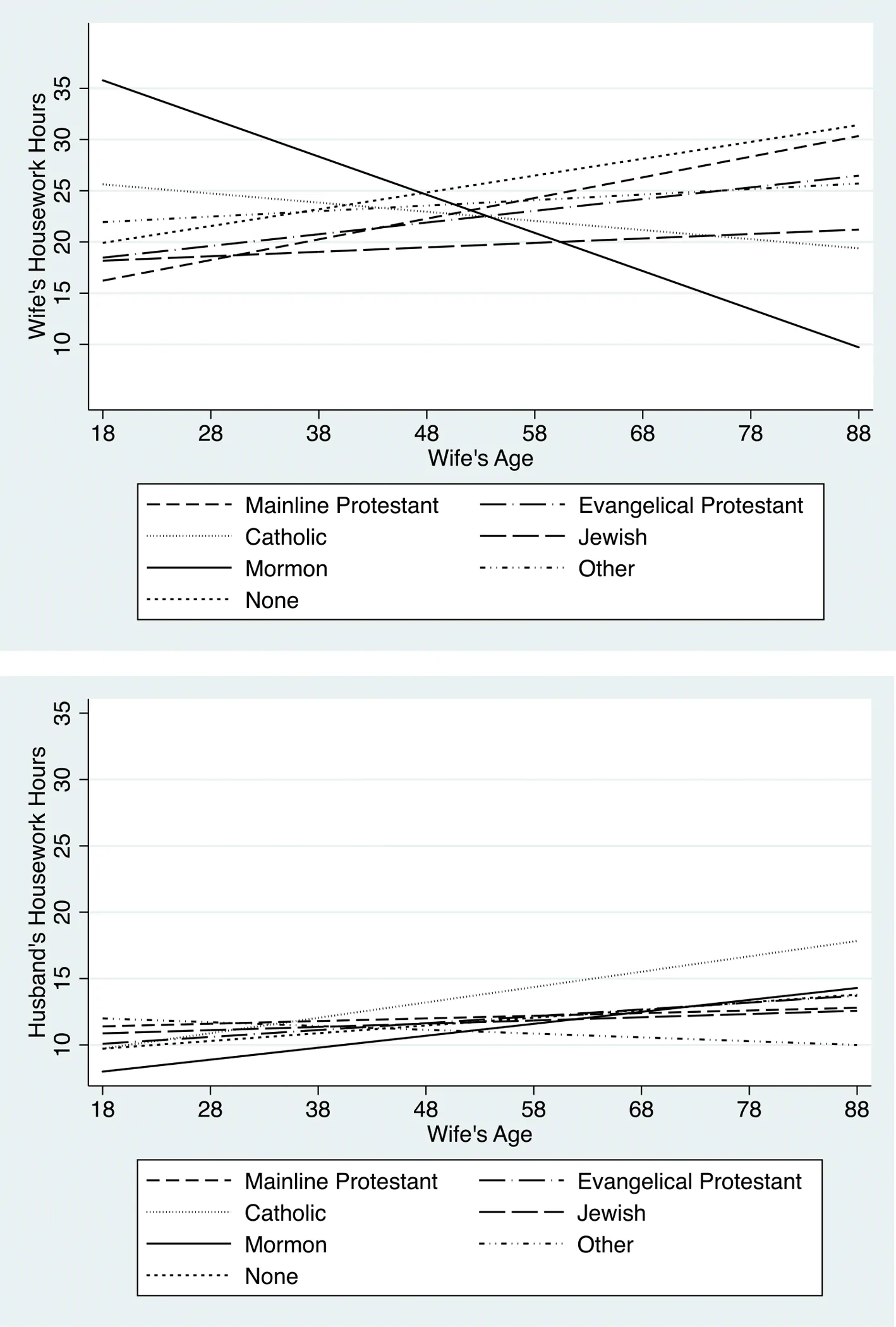
Predicted trajectories of wife’s and husband’s weekly housework hours by wife’s religious affiliation. Note: Predicted trajectories are based on estimates from Models 2a and 3a in Table 3. Source: Longitudinal Study of Generations (LSOG), 1988–2005.

Next, Models 2a and 3a (Table 3) analyze the interaction effect between wives’ religious affiliation and age on wives’ and husbands’ housework hours separately. Fig. 2 illustrates the interaction effects in these models. The results show that housework hours significantly decreased with age for Mormon wives (*b* = − 0.37, *p* < 0.01), while their husbands’ contributions remained stable over time. This suggests that the narrowing housework gender gap for Mormon wives over the life course may be primarily driven by reductions in their own housework time. In contrast, Catholic wives’ housework hours stayed consistent, but their husbands’ housework hours significantly increased with age (*b* = 0.12, *p* < 0.05) (results using Catholic as the reference category for Models 2a and 3a not shown). Thus, the declining housework gender gap for Catholic wives appears to stem from their husbands’ gradually increased housework contributions over the life course.

Paralleling the analysis of wives’ religious affiliation, we examined whether housework gender gap trajectories vary by husbands’ religious affiliation. Model 1f extends Model 1e by including the interaction between husbands’ religious affiliation and age (Table 3). Similar to the findings on wives’ religious affiliation, husbands’ religious affiliation was not significantly associated with the average housework gender gap at age 52. However, the significant interaction terms indicate that the slopes of housework gender gap trajectories differed by husbands’ religious denominations.

**Fig. 3.**
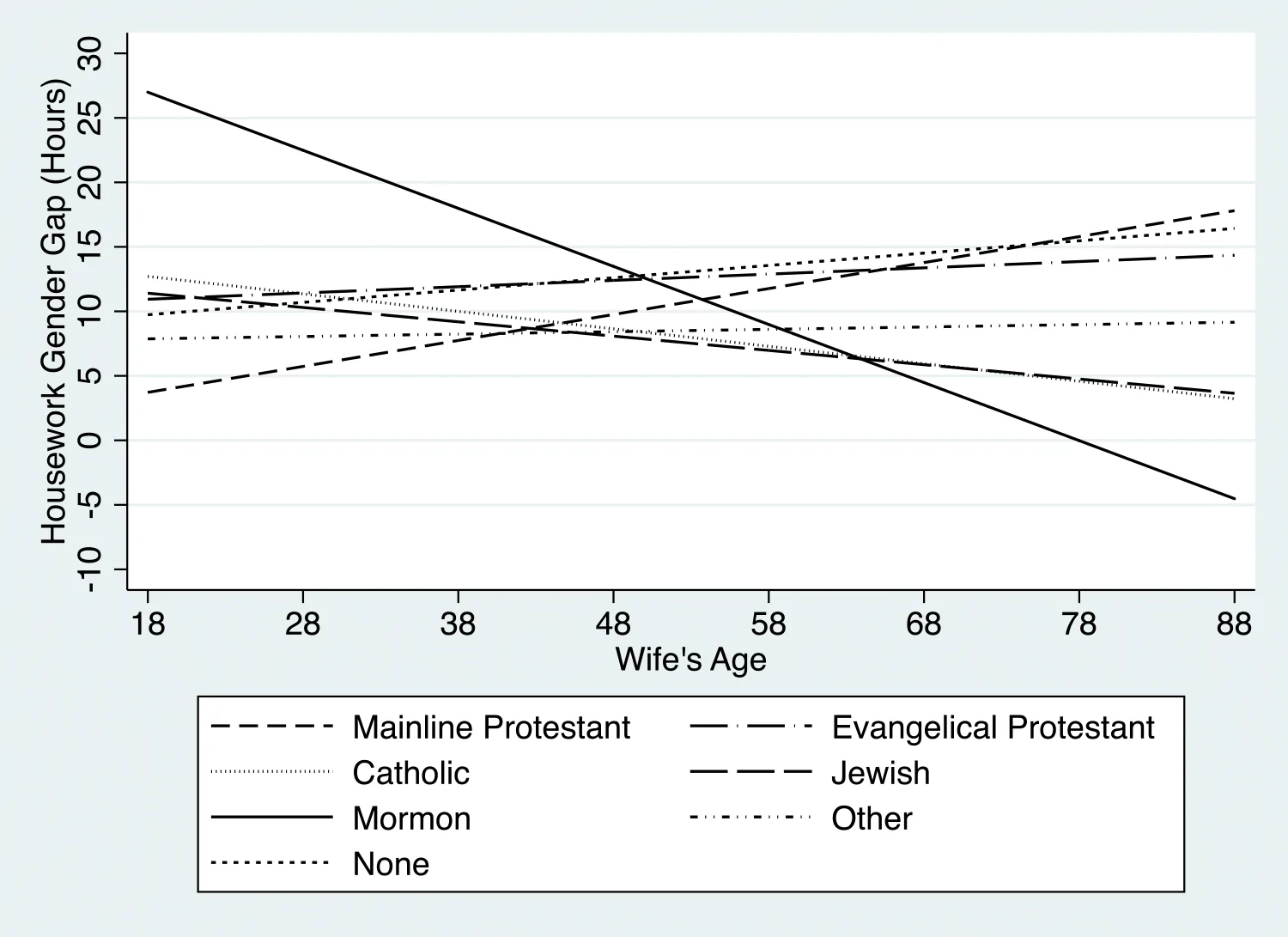
Predicted trajectories of housework gender gap by husband’s religious affiliation. Note: Predicted trajectories are based on estimates from Model 1f in Table 3. Source: Longitudinal Study of Generations (LSOG), 1988–2005.

Fig. 3 visualizes the interaction effects from Model 1f. The housework gender gap trajectory for Mormon husbands decreased significantly over the life course, at a rate of 0.45 h per year of age (Model 1f). In contrast, gender gaps in housework did not change significantly with age for husbands of any other religious affiliations or nonreligious husbands (analyses using each affiliation as the reference category not shown).

Additional tests at each age point in Fig. 3 revealed that disparities in the housework gender gap by husbands’ religious affiliation evolved with different patterns across life stages (results available upon request). In early life (ages 18–38), Mormon husbands exhibited significantly larger housework gender gaps than all other husbands. In midlife, no significant differences in the gender gap were observed across religious groups. In later life, Mormon, Catholic, and Jewish husbands exhibited significantly smaller housework gender gaps than mainline Protestant, evangelical Protestant, and nonreligious husbands. This pattern emerged around age 68 for Mormon husbands and between ages 48–58 for Catholic and Jewish husbands, persisting through age 88.

**Fig. 4.**
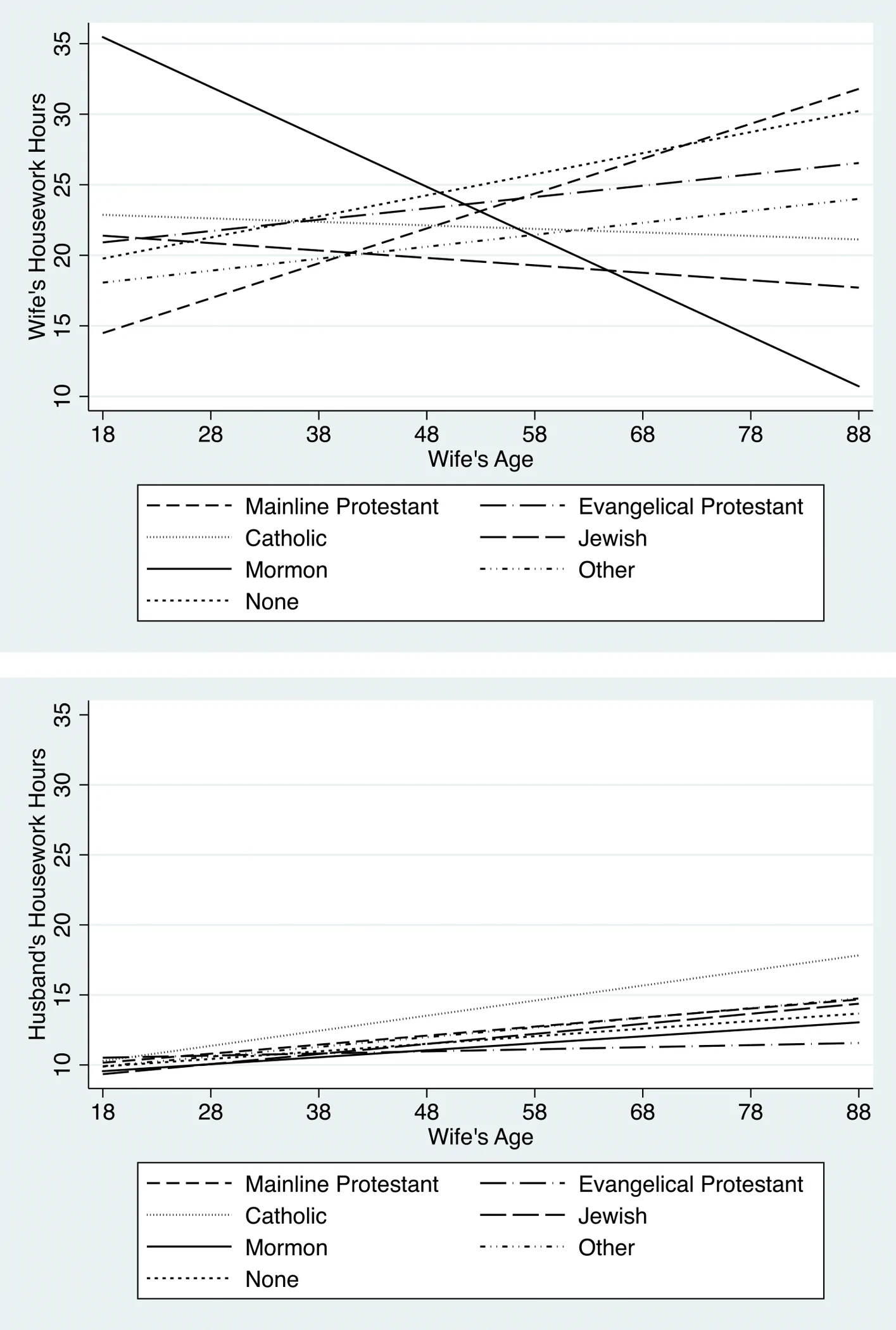
Predicted trajectories of wife’s and husband’s weekly housework hours by husband’s religious affiliation. Note: Predicted trajectories are based on estimates from Models 2b and 3b in Table 3. Source: Longitudinal Study of Generations (LSOG), 1988–2005.

Finally, Models 2b and 3b (Table 3) analyze the interaction effect of husbands’ religious affiliation and age using wives’ and husbands’ housework hours as the dependent variables. The interaction effects from these models are visualized in Fig. 4. The results indicate that among couples with Mormon husbands, wives’ housework hours significantly decreased with age (*b* = − 0.35, *p* < 0.05), while husbands’ housework hours remained stable over time. This suggests that the narrowing housework gender gap over the life course for Mormon husbands may be driven by reductions in their wives’ housework time, as their own contributions to housework did not change.

## 5. Discussion

Gender inequality in household labor remains a central topic in sociological research, with increasing attention to its persistence and changes over the life course. Taking a life course perspective, this study highlights the role of religious affiliations in shaping the within-couple housework gender gap as couples navigate different life stages. Our findings show that gender inequality in housework persists across nearly the entire adult life course, with wives consistently performing more housework than husbands, regardless of religious affiliation or nonreligious status. However, trajectories of the gender gap differ across some religious traditions, with wives’ and husbands’ religious affiliations similarly associated with these patterns.

Our findings partially support H1, as the housework gender gap declined significantly over the life course for couples where either spouse was Mormon or the wife was Catholic while remaining stable and broadly similar for others. Theological discourses and teachings in Mormonism and Catholicism emphasize conservative gender roles and traditional family values that prioritize women’s roles in homemaking and caregiving (Bulanda, 2011; Carroll et al., 2000; Heaton et al., 1994; Wilcox et al., 2004). In early life, when families typically expand with young children, the associated rise in housework demands may be primarily borne by wives of Mormon couples or Catholic wives due to theological justifications and strong normative pressures within close-knit religious communities, amplifying the gender gap in housework. Additionally, conservative religious identities, such as being Mormon, are closely tied to other family dynamics, including earlier marriage and parenthood, higher fertility, and reduced female labor force participation, particularly during childrearing years (Carroll et al., 2000; Glass & Nath, 2006; Heaton et al., 1994; Pew Research Center, 2015; Uecker & Stokes, 2008), further intensifying gender inequality in housework during early life.

The observed gender inequality in housework among Mormon couples and Catholic wives declined to levels comparable to other couples through midlife and continued to decrease in later life. This narrowing of the gender gap may be explained by shifting family dynamics that alter the demands and support for housework, leading to changes in spouses’ housework participation. Results on trajectories of wives’ and husbands’ housework time suggest that the decline in the housework gender gap for Mormon couples is likely due to a reduction in wives’ housework time over the life course rather than increased contributions from husbands, providing supporting evidence for H3. While this pattern may be partly explained by children growing older and leaving home, as seen in other religious and nonreligious groups (Craig & Sawrikar, 2009; Schulz & Raab, 2023), additional factors may be at play in Mormon families. Given their larger family sizes and early childbearing, older children – especially daughters – may help alleviate their mothers’ housework burden, having been socialized into similar gender and family roles. Parents may also encourage daughters to take on housework early as preparation for marriage and future family responsibilities. Additionally, social support from close ties within the religious community (Merino, 2014) that potentially includes assistance with household chores may further reduce women’s domestic workload.

Unlike Mormons, the decline in the housework gender gap for Catholic wives over the life course appears to stem from increased contributions to housework by husbands over time rather than a reduction in wives’ housework time, which aligns with H3. A possible explanation is the growing liberalization of gender and family attitudes among American Catholics, driven by rising socioeconomic status and interactions with liberal Catholics within the church, which may foster greater support for gender equality in family roles (Manning, 1997). Such social and institutional shifts likely encourage Catholic wives to seek and facilitate greater involvement in housework from their husbands, particularly when financial demands ease with children’s independence or retirement approaches.

Gender inequality in housework remained stable over the life course, with wives performing 5–15 more hours per week than husbands among Protestant (mainline or evangelical), Jewish, other religious, and nonreligious couples. Individuals in liberal religious traditions, such as mainline Protestant and Jewish, and those with no religious affiliation are more likely to hold egalitarian gender values (Fishman, 2014; Wilcox, 2004), fostering consistent housework-sharing practices despite fluctuations in housework demands across life stages. Among evangelical Protestants, the emergence of egalitarian strands within the church that embrace flexible gender roles, along with an increasing theological emphasis on men’s greater familial involvement (Civettini & Glass, 2008; Ellison & Bartkowski, 2002), may have helped maintain rather than widen the housework gender gap, even during the most demanding parenting years. The stable trajectories observed in these groups suggest that once relatively egalitarian housework division norms and practices are established, they tend to persist and reinforce relatively smaller gender gaps as couples navigate new life transitions.

Finally, comparing trajectories across religious groups, religious disparities in the housework gender gap follow distinct patterns at different life stages, supporting H2. In early life, gender inequality in housework was highest among Mormon couples, followed by Catholic wives, while couples in other religious and nonreligious groups exhibited lower and similar levels of inequality. These findings align with cross-sectional research indicating greater gender inequality in housework among Mormons and Catholics (Heaton et al., 1994; Voicu et al., 2009). During midlife, no significant religious differences in housework gender inequality were observed, suggesting a convergence across religious groups. However, in later life, gender inequality in housework was lower among Mormon, Catholic, and Jewish couples than among their Protestant (mainline or evangelical) and nonreligious counterparts. These results extend prior cross-sectional findings of minimal differences in housework division between conservative and mainline Protestants (Civettini & Glass, 2008; Ellison & Bartkowski, 2002) across the life course. Theological developments and ideological shifts toward more flexible gender roles and greater male involvement in family responsibilities have gained traction among conservative Protestants (Civettini & Glass, 2008; Ellison & Bartkowski, 2002). Despite affiliating with conservative religious traditions, many couples adopt pragmatic egalitarianism in practice or embrace more flexible and diverse attitudes toward gender and family roles (Bulanda, 2011; Gull & Geist, 2020), which may potentially blur distinctions in housework behavior between conservative and mainline Protestants. Additionally, the low salience of religious differences in the housework gender gap in midlife and among some groups at other life stages suggests that time availability and economic resources – significant controls in the models – may play a greater role in shaping gender inequality in housework.

The substantial gender inequality faced by Mormon couples and Catholic wives in early life, along with the persistent yet moderate gender inequality in housework for most wives regardless of religious affiliation, has significant implications. According to cumulative disadvantage theory (O’Rand, 2006), disadvantages brought by enduring demands of housework or the double burden of work and domestic responsibilities can accumulate over time, leading to broader disadvantages for women in later life. Women experiencing persistent housework inequality may have intermittent careers, reduced work hours, and lower earnings (Perry-Jenkins & Gerstel, 2020), leading to widening gender disparities in resource accumulation, retirement pathways, and financial independence in later life (Moen, 2016). Housework, particularly tasks traditionally performed by women, can be overwhelming, unappreciated, and isolating. The strain of housework overload or the double burden of work and domestic labor negatively affect women’s physical and mental well-being (Pinho & Araújo, 2012; Roxburgh, 2004; Strazdins et al., 2016). When these burdens persist, their cumulative toll can contribute to widening health disparities in later life (Chen et al., 2019). Mormon women report lower happiness and higher depression than their non-Mormon counterparts (Heaton et al., 1994), which may, in part, reflect the burden of their larger housework share in early life.

Several limitations should be noted when interpreting these findings. First, the survey measured the total hours of household chores without distinguishing between specific tasks. This may introduce greater measurement error than task-specific estimates. Direct survey questions also tend to overestimate housework hours for both wives and husbands compared to time diary data (Coltrane, 2000). Second, the data do not separate stereotypically feminine housework tasks from others, preventing an analysis specific to those tasks. While many studies focus on gender gaps in traditionally feminine tasks, survey-based measures that include all housework tasks are also widely used in research on gender inequality in household labor (Bernhardt et al., 2008; Bianchi et al., 2000; Voicu et al., 2009). Although a single question on overall housework time is easier to administer repeatedly in longitudinal surveys, it requires caution in interpretation, as a significant portion of men’s reported housework may include traditionally masculine tasks. As a result, our measure of the gender gap may underestimate inequality in traditionally feminine tasks, which are often more regular, time-intensive, monotonous, and subject to rigid schedules (Cunningham, 2005). Third, generalizability should be considered with caution for several reasons. The survey initially sampled predominantly White, middle-class families in California, though later waves became more diverse. Additionally, while our study spans 17 years, the last wave was collected in 2005, which may limit the applicability to more recent periods. Future research incorporating more recent and nationally representative data can further examine these trends. Finally, our findings from growth curve models are associational rather than causal, requiring cautious interpretations. The models cannot disentangle age, period, and cohort (APC) effects due to collinearity with calendar year. While the observed trends likely reflect life course processes, they may also capture broader societal changes or cohort differences. Future research can apply APC models to isolate these effects. Furthermore, although higher-order age terms were tested and found to be non-significant, assuming linear change may oversimplify life course patterns. Future studies can benefit from alternative approaches, such as spline regressions, to capture more flexible temporal trends.

Despite these limitations, this study advances the understanding of gender inequality in housework over the life course by highlighting how such trajectories differ across religious affiliations. The findings inform targeted interventions to prevent cumulative disadvantages from excessive housework in early life. Support programs aimed at early life stages that promote women’s economic independence and well-being may help mitigate inequalities later in life. Religious disparities in gender inequality in housework exhibit different patterns across life stages, emphasizing the importance of taking a life course perspective to situate the relationship between religion and housework in social, economic, and cultural contexts that shape family dynamics and housework at each life stage and religion’s interplay with them. Future research could further investigate the mechanisms through which religion influences housework over the life course and extend this inquiry to childcare and cognitive labor across more diverse populations in terms of race/ethnicity, social class, and sexual orientation.

## Figures and Tables

**Fig. 1.**
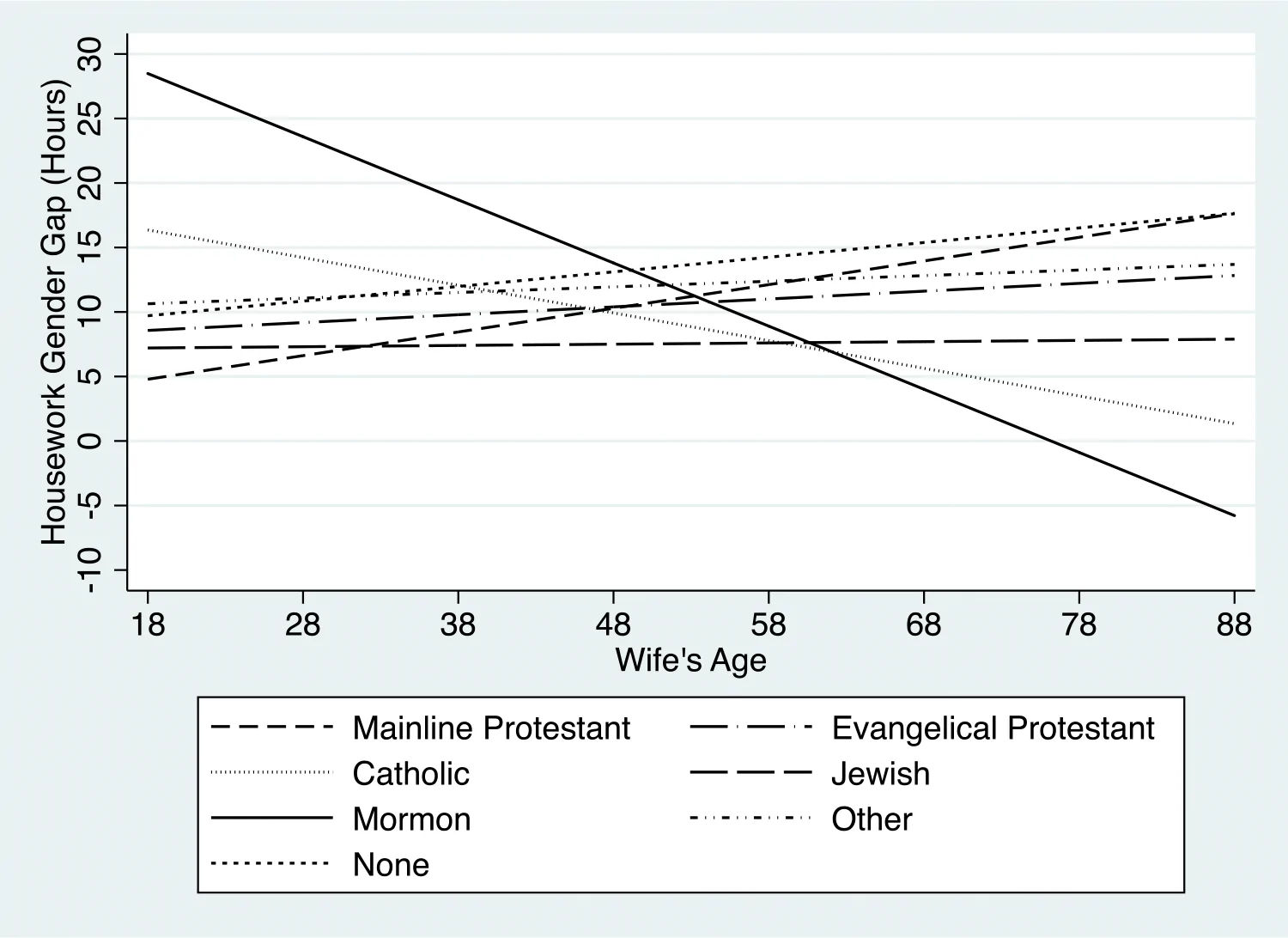
Predicted trajectories of housework gender gap by wife’s religious affiliation. Note: Predicted trajectories are based on estimates from Model 1d in Table 3. Source: Longitudinal Study of Generations (LSOG), 1988–2005.

**Table 1:** Descriptive statistics of variables in the analyses (n = 1487).

	Mean		Mean

**Dependent Variables**		Wife’s self-reported health	
Housework gender gap	11.31	Poor/fair (Ref. Cat.)	0.19
Wife’s weekly housework hours	23.22	Good	0.54
Husband’s weekly housework hours	11.90	Excellent	0.27
**Independent Variables**		Husband’s self-reported health	
Wife’s age	48.99	Poor/fair (Ref. Cat.)	0.18
Wife’s religious affiliation		Good	0.51
Mormon (Ref. Cat.)	0.07	Excellent	0.30
Mainline Protestant	0.19	Wife’s education	
Evangelical Protestant	0.18	High school or less (Ref. Cat.)	0.21
Catholic	0.16	Some college	0.43
Jewish	0.08	College degree	0.18
Other	0.03	Graduate degree	0.18
None	0.30	Husband’s education	
Wife’s religious attendance		High school or less (Ref. Cat.)	0.16
Infrequent (Ref. Cat.)	0.46	Some college	0.33
Moderate	0.19	College degree	0.20
Frequent	0.35	Graduate degree	0.31
Husband’s religious affiliation		Wife’s income last year	2.27
Mormon (Ref. Cat.)	0.07	Husband’s income last year	4.77
Mainline Protestant	0.16	Number of children in the household	0.89
Evangelical Protestant	0.16	Wife’s work hours per week	23.26
Catholic	0.13	Husband’s work hours per week	36.49
Jewish	0.09	Wife’s gender ideology	2.99
Other	0.03	Husband’s gender ideology	2.85
None	0.37	Loss to follow-up	
Husband’s religious attendance		No (Ref. Cat.)	0.80
Infrequent (Ref. Cat.)	0.50	Yes	0.20
Moderate	0.18	Divorced/separated	
Frequent	0.32	No (Ref. Cat.)	0.93
**Control Variables**		Yes	0.07
Wife’s race/ethnicity		Widowed	
Other races/ethnicities (Ref. Cat.)	0.07	No (Ref. Cat.)	0.95
White	0.93	Yes	0.05
Husband’s race/ethnicity			
Other races/ethnicities (Ref. Cat.)	0.08		
White	0.92		

Source: Longitudinal Study of Generations (LSOG), 1988–2005.

**Table 2:** Growth curve models predicting the gender gap in weekly housework hours for couples.

	Model 1a Housework gender gap	Model 1b Housework gender gap
Fixed effects		
Intercept	10.97 *** (0.68)	13.62 ** (4.85)
Wife’s age (grand mean-centered)	− 0.13 ** (0.04)	− 0.01 (0.05)
**Control Variables**		
Wife’s race/ethnicity (Ref. Cat. = Other races/ethnicities)		
White		− 0.21 (2.64)
Husband’s race/ethnicity (Ref. Cat. = Other races/ethnicities)		
White		1.79 (2.57)
Wife’s self-reported health (Ref. Cat. = Poor/fair)		
Good		2.32 (1.39)
Excellent		2.83 (1.62)
Husband’s self-reported health (Ref. Cat. = Poor/fair)		
Good		0.62 (1.45)
Excellent		− 0.11 (1.68)
Wife’s education (Ref. Cat. = High school or less)		
Some college		− 1.36 (1.50)
College degree		− 2.51 (2.03)
Graduate degree		− 3.05 (2.12)
Husband’s education (Ref. Cat. = High school or less)		
Some college		− 0.69 (1.72)
College degree		− 1.24 (2.06)
Graduate degree		− 1.28 (2.06)
Wife’s income last year		− 1.38 *** (0.29)
Husband’s income last year		0.44 * (0.21)
Number of children in the household		3.40 *** (0.56)
Wife’s work hours per week		− 0.07 * (0.03)
Husband’s work hours per week		0.11 ** (0.04)
Wife’s gender ideology		− 1.51 (1.21)
Husband’s gender ideology		− 1.18 (1.19)
Loss to follow-up (Ref. Cat. = No)		
Yes		− 1.04 (1.45)
Divorced/separated (Ref. Cat. = No)		
Yes		0.13 (2.29)
Widowed (Ref. Cat. = No)		
Yes		4.48 (2.55)
Random effects - variance components		
Level 1 (wave-specific): within-couple	281.30 (14.42)	280.12 (14.61)
Level 2 (couple-specific): In intercept	106.85 (20.92)	59.56 (17.31)
Level 2 (couple-specific): In linear growth rate	0.25	0.19
n of couple dyads	(0.08) 661	(0.07) 661
n of dyad-year observations	1487	1487

Notes:

* p < .05,

** p < .01,

*** p < .001. All random effects were statistically significant based on estimates of their 95 % confidence intervals.

Source: Longitudinal Study of Generations (LSOG), 1988–2005.

**Table 3:** Growth curve models predicting the effect of religion on the gender gap and weekly housework hours for couples.

	Model 1c Housework gender gap	Model 1d Housework gender gap	Model 2a Wife’s housework hours	Model 3a Husband’s housework hours

Intercept	13.98 * (5.62)	12.23 * (5.63)	29.36 *** (5.10)	16.43 *** (2.79)
Wife’s age (grand mean-centered)	0.00 (0.05)	− 0.49 ** (0.15)	− 0.37 ** (0.13)	0.09 (0.08)
Wife’s religious affiliation (Ref. Cat. = Mormon)				
Mainline Protestant	− 2.81 (2.85)	− 0.87 (2.87)	− 0.08 (2.64)	1.03 (1.54)
Evangelical Protestant	− 3.43 (2.77)	− 1.24 (2.84)	− 0.80 (2.62)	0.80 (1.55)
Catholic	− 4.52 (2.89)	− 2.80 (2.92)	− 0.55 (2.70)	2.61 (1.59)
Jewish	− 6.38 (3.43)	− 4.34 (3.44)	− 3.50 (3.16)	0.65 (1.80)
Other	− 1.79 (4.10)	0.24 (4.10)	0.62 (3.67)	− 0.02 (2.36)
None	− 0.51 (2.94)	1.68 (2.96)	2.34 (2.72)	0.65 (1.59)
Mainline Protestant × Wife’s age		0.67 *** (0.18)	0.57 *** (0.16)	− 0.07 (0.09)
Evangelical Protestant × Wife’s age		0.55 ** (0.17)	0.49 ** (0.15)	− 0.04 (0.09)
Catholic × Wife’s age		0.28 (0.18)	0.28 (0.16)	0.03 (0.10)
Jewish × Wife’s age		0.50 * (0.21)	0.42 * (0.19)	− 0.07 (0.11)
Other × Wife’s age		0.53 * (0.23)	0.43 * (0.20)	− 0.12 (0.14)
None × Wife’s age		0.60 *** (0.17)	0.54 *** (0.15)	− 0.03 (0.09)
Wife’s religious attendance (Ref. Cat. = Infrequent)				
Moderate	0.68 (1.56)	0.61 (1.55)	0.36 (1.40)	− 0.60 (0.73)
Frequent	1.76 (1.67)	1.75 (1.67)	1.43 (1.52)	− 0.85 (0.81)
n of couple dyads	661	661	661	661
n of dyad-year observations	1487	1487	1487	1487

	Model 1e Housework gender gap	Model 1f Housework gender gap	Model 2b Wife’s housework hours	Model 3b Husband’s housework hours

Intercept	16.00 ** (5.66)	12.66 * (5.73)	30.36 *** (5.23)	16.03 *** (2.82)
Wife’s age (grand mean-centered)	0.01 (0.05)	− 0.45 ** (0.16)	− 0.35 * (0.14)	0.05 (0.08)
Husband’s religious affiliation (Ref. Cat. = Mormon)				
Mainline Protestant	− 3.49 (3.00)	− 1.17 (3.10)	− 0.60 (2.87)	1.10 (1.60)
Evangelical Protestant	− 1.86 (2.96)	0.87 (3.08)	0.17 (2.85)	− 0.22 (1.61)
Catholic	− 6.11 * (3.09)	− 3.61 (3.17)	− 1.45 (2.93)	2.70 (1.65)
Jewish	− 6.81 * (3.42)	− 4.07 (3.50)	− 3.86 (3.26)	0.54 (1.78)
Other	− 5.91 (4.23)	− 3.23 (4.28)	− 2.53 (3.97)	1.01 (2.23)
None	− 1.53 (3.11)	1.26 (3.21)	1.37 (2.96)	0.48 (1.63)
Mainline Protestant × Wife’s age		0.65 *** (0.18)	0.60 *** (0.16)	0.01 (0.09)
Evangelical Protestant × Wife’s age		0.50 ** (0.18)	0.43 ** (0.16)	− 0.03 (0.09)
Catholic × Wife’s age		0.31 (0.19)	0.33 (0.17)	0.06 (0.10)
Jewish × Wife’s age		0.34 (0.21)	0.30 (0.19)	0.02 (0.11)
Other × Wife’s age		0.47 (0.26)	0.44 (0.23)	0.02 (0.14)
None × Wife’s age		0.55 ** (0.17)	0.50 *** (0.15)	0.00 (0.09)
Husband’s religious attendance (Ref. Cat. = Infrequent)				
Moderate	− 3.36 * (1.67)	− 3.44 * (1.66)	− 2.28 (1.51)	1.05 (0.77)
Frequent	− 0.83	− 0.54	− 0.80	− 0.28
	(1.82)	(1.83)	(1.66)	(0.89)
n of couple dyads	661	661	661	661
n of dyad-year observations	1487	1487	1487	1487

Notes:

* p < .05,

** p < .01,

*** p < .001. All models also estimated fixed effects of all control variables from Table 2 as well as random effects. Full results are available upon request.

Source: Longitudinal Study of Generations (LSOG), 1988–2005.

**Fig. 1.**
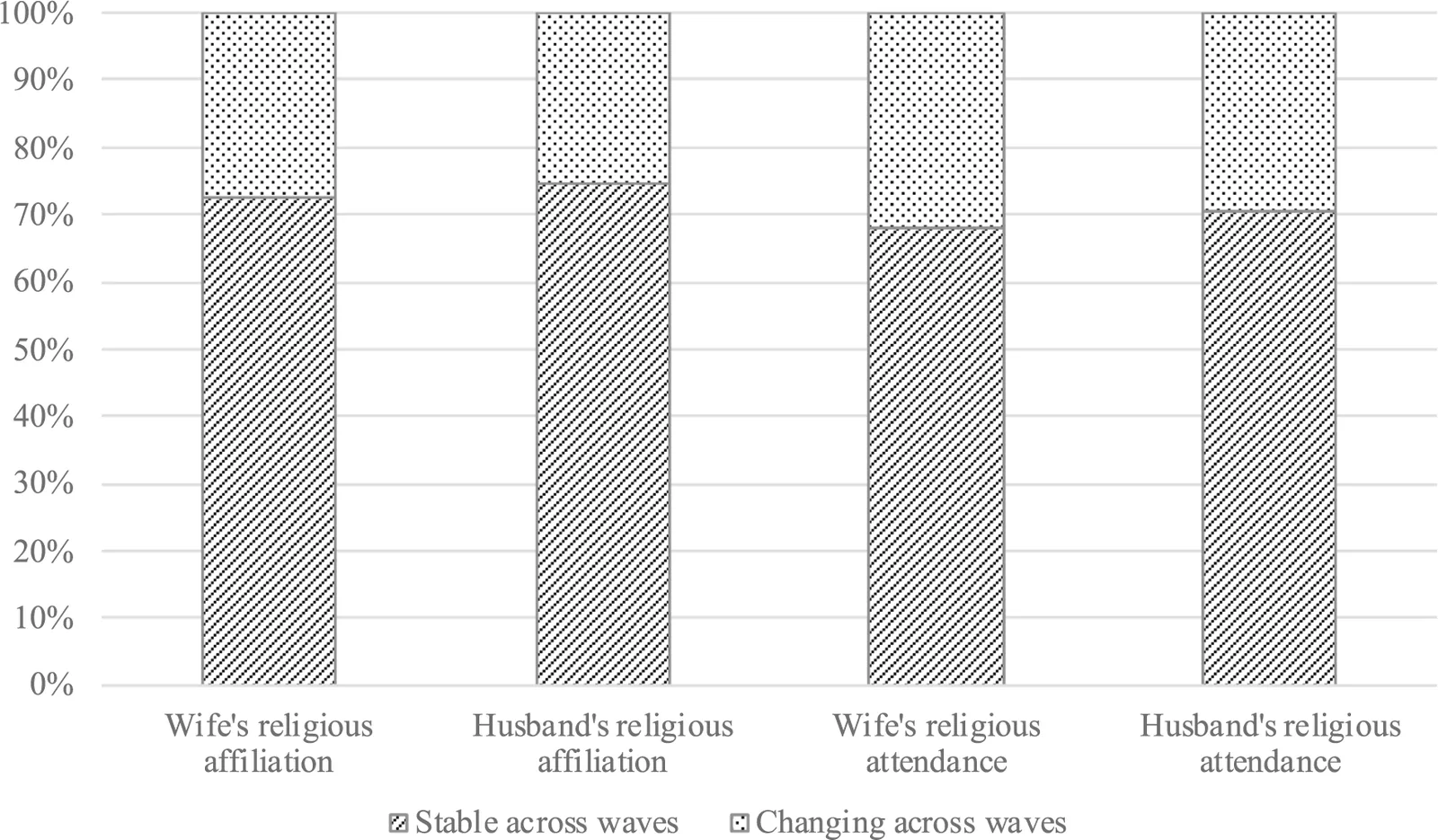
Within-couple stability and change in religious characteristics among couple dyads interviewed in at least two waves (n = 399). Source: Longitudinal Study of Generations (LSOG), 1988–2005.
